# Risk factors for alcohol and other drug use by healthcare professionals

**DOI:** 10.1186/1747-597X-3-3

**Published:** 2008-01-29

**Authors:** George A Kenna, David C Lewis

**Affiliations:** 1 Center for Alcohol and Addiction Studies, Brown University, Box G-121S-4, Providence, RI 02908, USA

## Abstract

**Background:**

Given the increasingly stressful environment due to manpower shortages in the healthcare system in general, substance induced impairment among some healthcare professions is anticipated to grow. Though recent studies suggest that the prevalence of substance abuse is no higher in healthcare professionals (HPs) than the general population, given the responsibility to the public, any impairment could place the public at increased risk for errors. Few studies have ever reported predictors or risk factors for alcohol and other drug use (AOD) across a sample of HPs.

**Methods:**

The study used a cross-sectional, descriptive self-report survey in a small northeastern state. A 7-page survey was mailed to a stratified random sample of 697 dentists, nurses, pharmacists and physicians registered in a northeastern state. The main outcome measures were demographic characteristics, lifetime, past year and past month prevalence of AOD use, the frequency of use, drug related dysfunctions, drug misuse and abuse potential. Six contacts during the summer of 2002 resulted in a 68.7% response rate (479/697).

**Results:**

Risk factors contributing to any reported past year AOD use, as well as significant (defined as the amount of AOD use by the top 25% of respondents) past year AOD use by HPs were examined using logistic regression. Risk factors of any self-reported past year AOD use included moderate or more frequency of alcohol use, being in situations when offered AODs, feeling immune to the addictive effects of drugs (pharmaceutical invincibility) and socializing with substance abusers. Risk factors of significant past year AOD use were HPs with younger licensees, a moderate pattern of alcohol use and not socializing with substance abusers.

**Conclusion:**

National and state organizations need to develop policies that focus on prevention, treatment, and rehabilitation of alcohol and other drug-using healthcare professionals. The results of this study may help to delineate the characteristics of HPs abusing drugs, leading to the development of more effective policies designed to protect the public, and move toward more tailored and effective intervention strategies for HPs.

## Background

The pathways toward development of substance abuse and dependence problems in healthcare professionals (HPs) vary by group. For example, though professionally discouraged, self-diagnosing physicians have reported prescribing controlled substances for themselves [1]. Due to drug access, a significant number of pharmacists tend to self-medicate and have the opportunity to titrate their drug use [2], a practice that can perpetuate the fallacy that pharmacological knowledge of drug action is an effective strategy to prevent addiction. In addition to drug access and a social environment promoting drug use [3] people who choose nursing as a profession may report a higher rate of family history of alcoholism and drug abuse than other HP groups [4]. Finally, perhaps more so than any other group of HPs, the greatest threat in dentistry may be alcohol consumption not controlled substance use [5]. Given the increasingly stressful environment due to manpower shortages in the healthcare system in general, substance induced impairment among some healthcare professions is anticipated to grow [6]. While a number of studies provide some foundation for our understanding of the epidemiologic and etiologic processes contributing to substance use by HPs, rarely have recent studies sought to examine the relative importance of risk factors on alcohol and drug (AOD) use across a sample of major groups of HPs. The major aim of this study therefore was to investigate the risk factors contributing to AOD use by HPs within the context of self-reported past year alcohol and drug use at different levels of use: any alcohol and drug use and a substantial level of alcohol and drug use. Early identification is essential as patient and provider well-being may be at risk [7]. Just like evidence for alcohol [8], cocaine [9], and illicit drugs [10], drug dependent HPs represent a specific subtype of drug abuser who have access to licit drugs. Knowing those HPs who may be at greatest risk for substance abuse, may facilitate the development of more tailored and effective educational and intervention strategies for this subtype of addict.

## Method

### Sample

The study was approved by the University of Rhode Island Institutional Review Board. Guidelines for sampling were based on each profession's size in the targeted population: a northeastern state [11]. The sample was drawn from lists for each profession supplied by the state Department of Health during 2002 and was stratified by zip codes to be representative of all regions of the state. All HPs currently licensed by and living in the surveyed state, as well as two closely adjoining states, were eligible.

Based on the population of each HP group in the state, random sampling of HPs consisted of a process of choosing every "n^th^" person on each group's list. The sample frame was 671 for dentists, 1369 for pharmacists, 3424 for physicians and 15,181 for nurses. The total anticipated to obtain the targeted final sample of HCPs was 748, consisting of 178 dentists, 188 nurses, 186 pharmacists and 196 physicians (95% confidence level with ± 10% sampling error).

### Survey Administration

During the summer of 2002, using validated self-report mail survey methods [11] participants chosen from the sample frame were sent an introductory letter explaining the purpose of the survey. Beginning at regular, specified intervals over an eight-week period, participants were mailed a consent form, cover letter, seven-page survey, materials to return the survey and to maintain confidentiality – a postcard to be mailed separately from the survey to remove participants from future contacts. As a token of appreciation, one dollar was sent to all participants in the first wave of surveys. After the introductory letter, receipt of three surveys and two follow-up postcards were possible, with subsequent mailings going only to non-respondents from previous mailings. The final survey mailed to the remaining non-respondents was made by priority mail.

### The Questionnaire

The items used in the current study and part of a larger survey, drew on earlier surveys of HPs [12,13]. A set of 12 items assessed demographic information including profession, area and specialty of practice, gender, race, ethnicity, current employment, income, marital status and age.

Categorizations delineated below were based on those used in previous studies and were retained for comparability [12,13]. Respondents were asked to report their regular number of drinks per day consumed and number of days per week of alcohol use during the past year and extrapolated into a 30-day alcohol use index.

Substances assessed in addition to alcohol included, tobacco (quantity and frequency), legal medications such as stimulants (e.g. amphetamine and methylphenidate), major opiates (e.g. methadone, hydromorphone, fentanyl, meperidine, morphine, and oxycodone), minor opiates (e.g. hydrocodone, pentazocine and codeine), anxiolytics (e.g. alprazolam), sedative-hypnotics (e.g. zolpidem and temazepam), inhalants (e.g. nitrous oxide and amyl nitrate), and tranquilizers (e.g. phenobarbital and ketamine). Two drugs, heroin and steroids, assessed in much larger samples have provided little data in view of very low base rates [13] and were not assessed. To provide general familiarity, trade names rather than generic terms were used as examples of drugs within each drug class. "Street" drugs assessed included marijuana, cocaine (composite analyses considered cocaine a prescription drug), psychedelics (e.g. lysergic acid diethylamide, mescaline and phencyclidine), designer drugs (e.g. methylenedioxymethylamphetamine, gamma hydroxybutyrate, rohypnol, and methamphetamine). Regular use for a drug other than alcohol was defined as use greater than 11 times during the past year or once per month or more [1].

#### Pattern of alcohol use

Consistent with previous research, one question assessing type of drinking pattern asked, "I consider myself a:" Responses were, "non-drinker," "infrequent drinker," "light drinker," "moderate drinker," "heavy drinker," and "problem drinker."

#### Family history of alcohol and drug use

A set of two items were hypothesized to measure family history of alcohol and drug abuse. These measures were adapted from the short MAST [14] and were previously reported in greater detail [15]. In addition to no family members affected, information regarding alcohol and drug abuse problem-history was requested for eight close family members: father, mother, sister, brother, grandfather, grandmother, uncle and aunt [16].

#### Professional invincibility

One item was hypothesized to measure attitudes towards one's ability to self-medicate without becoming addicted, for example the question asked "Please indicate your degree of confidence that your pharmacotherapeutic (pharmacodynamic and pharmacological) knowledge assures that you would never become addicted to medications or drugs." Response options consisted of a six-point Likert scale from "Not confident at all" to "Totally confident" and "Undecided" (not included in analysis). Two other items assessed how strongly participants believed self-medication to stay on the job, or function at home, was an acceptable practice. For example one question asked, "Please indicate how strongly do you agree or disagree that it is appropriate to take non-prescribed controlled prescription medication to function at work?" (e.g., you have a bad cough and take a codeine type cough syrup, or hurt your back and take a narcotic analgesic to keep working etc.). A six-point Likert scale provided response options. Coefficient alpha was .23 for these three items. Eliminating one variable (item 1) would increase α to a maximum of .31. DeVellis [17], Stevens [18] and Comrey [19] suggest a minimum alpha of .6 to .8. Furthermore, as recommended by Clark and Watson [20] the inter-class correlations could not pass the .15 barrier that they recommend. While the scale was not used – an individual item (the first) was used in the logistic regression analysis.

#### Internal religiosity

Internal religiosity assessed the internal aspect of negative proscriptions related to religion [3,21], and was a set of five items. Internal consistency for this scale was α = .89. For example, one question asked, "Religion gives me a great amount of comfort and security in life." Respondents scoring highly on internal religiosity would be expected to use less if any drugs.

#### Social networks

Adapted from Trinkoff et al. [3], this set of three questions assessed the external aspect of negative proscriptions and asked, "Do you personally know anyone, (other than patients or clients) who (a) drinks heavily or has an alcohol problem (b) uses illicit street drugs or (c) uses prescription drugs on his/her own?" Responses for each question included "spouse or partner," "friend," "coworker or colleague," or "other." Two experts in the Trinkoff et al. [3] study validated the original items for content, however no internal consistency data were provided. As family histories of alcohol and drug use were assessed separately, "parent" was deleted as a response from this measure. Respondents were asked to check all that apply. The range of scores were from 0 (no substance users in social network) to 3 (know someone who uses all three categories of substances).

#### Drug access

Drug access was operationalized as workplace access to controlled substances. Four questions assessed the availability of Class II through V controlled substances, non-controlled prescription medications and of street drugs. Brand names of drugs for recognition purposes were used when possible. For example, participants were asked to respond to the phrase "During the past year: if I really wanted to get C-2 medications (e.g. Percocet, fentanyl, morphine, Oxycontin etc.) inappropriately I could have." A Likert scale with response options ranging from Strongly disagree to Strongly agree was provided. Coefficient alpha for these four items was .82.

#### Negative proscriptions

An index was made comprised of religiosity, social networks and drug access [3]. Internal religiosity was recoded so that higher scores reflected lower religiosity. The three items were then summed for an index. Consistent with Trinkoff et al. [3] higher scores on this index would be at greater risk for drug use than lower scores.

#### Protective beliefs

Consistent with previous research in this area [22,23], four items generated by the author considered how strongly participants feared either the ethical, legal, social, or moral repercussions of being caught inappropriately using medications or drugs, or a participants fear of physical-mental reactions to taking mediations or drugs. For example one question asked, "Do you agree or disagree that you fear the legal repercussions of being caught inappropriately using medications or drugs." Coefficient alpha for this scale was .94.

#### Professional and social influences

A set of three questions assessed the impact of "active" social influences on alcohol and illicit use of legal medications. Two of these items were hypothesized to measure these "active" social influences. These are situations in which offers of alcohol are made by friends or colleagues (items adapted from Graham et al [24], Wood et al. [25], and seminars offering alcohol supported by pharmaceutical companies and a decision to accept or deny the offer is made spontaneously. In addition, one question assessed the effects of active social influences involved with the illicit use of legal medications [22]. In general, participants were asked to respond with regard to how many times in the past year they had been in situations in which they were either given, bought or offered either a drink by friends and colleagues or salespeople, or offered prescription substances without asking for them. Specifically one question asked for example, "How many times in the past year has a friend given, bought, or offered you an alcoholic beverage." Respondents were provided five-point Likert response options ranging from "Never" to "10 or more times." Four questions of interest assessed professional influences and were based on a question used previously [26], with additional items generated by the researcher, and were designed to assess peripheral professional job-related social influences during the past year. An example of one question was, "How many times during the past year have you been asked for psychoactive prescriptions (or medications) by colleagues or non-patients?" Coefficient alpha for the seven items from the social and professional subscales was .71. A separate scale removing the two alcohol questions resulted in an alpha = .62.

### Data Analyses

Data analyses were carried out using the Statistical Analysis System (SAS Institute, Cary, NC). The initial phase consisted of distribution analyses and variable formation for further analysis. With the exception of demographics or discrete variables, mean substitution was used to replace missing values [27] as standard deviations were ubiquitously small (SD < 1.0). Variables used in analyses in this study met guidelines for skew and kurtosis [27].

Because the survey was returned anonymously there was no way to examine direct differences between those who responded and those who did not. However, to assess response bias and if there was any relationship between various substance use measures and when (which wave) the survey was returned, the data were examined using logistic regression, analyses of variance (ANOVA) and bivariate correlations for lifetime drug use as a function of wave of data. These methods assume that wave three responders are more characteristic of non-responders than are those respondents to the first wave of surveys. There would be a bias toward lower estimates of substance use if that were the case. In other words, if substance users were less likely to respond, then the third wave respondents would have a higher proportion of substance users[28,29]. First, logistic regression was used to assess the adjusted odds ratios of several predictor variables regressed on early responders from wave one of the survey versus late responders first from wave two then wave three assuming good model fit [30]. The variables used in the models were total weekly alcohol use, lifetime frequency of occasions to drink five or more alcoholic drinks at one time, lifetime use of minor opiates, gender, alcohol and drug problems, treatment, and active practice of a religion were regressed on early versus late responders. The variables chosen represented significant reported HP behaviors of interest in this study. Analyses showed that there was a lack of model fit criteria, suggesting that these variables were not good predictors of early versus late return. Next, a series of wave by ANOVAs were conducted on these same measures. These analyses revealed there were no significant differences of the occurrences in any of these variables by wave. Finally, when the survey was returned (wave) was examined as a bivariate correlation with lifetime drug use episodes that were re-coded as the median for each response category and then summed (with a value of 75 times assigned to each drug use category of ">61.") For all HPs combined, the correlation between wave the survey was returned and drug use was non-significant (r = .01). For dentists, while non-significant (p = .08), there was a trend that number of episodes of drug use was associated with a earlier response (r = -.17). For nurses, pharmacists and physicians, the number of episodes of drug use was non-significantly correlated with a later response (r = .14, p = .11), (r = .10, p = .27) and (r = -.07, p = .49) respectively. Despite the poor model fit associated with the logistic regression analyses, the other methods employed in the current study found little evidence for systematic non-response bias in these data.

To examine the major aim of the study, logistic regression was used to assess the biopsychosocial factors predicting past year AOD use. The biopsychosocial model is a theory that biological, psychological, and social factors all play significant roles in human functioning in disease or illness, and that substance abuse, in this case, is a result of the combination of these factors [31]. The model proposes that biological, psychological, and sociological spectrums are interconnected. The model represents a dramatic shift in focus from disease to health, recognizing that psychosocial factors (e.g. beliefs, relationships, stress) greatly impact illness and disease. More specifically, the biopsychosocial model suggests that addiction is a brain disease that may cause personality problems and social dysfunction and furthermore delineates a clear and accurate distinction between substance use, abuse, and addiction. It also allows the progressive symptoms of addiction to be readily identified and organized into progressive stages [31].

Assessed risk factors consisted of biological (family history of substance use), demographic (gender, age), occupational (drug access) and legal drug use (current cigarette use). Social factors included exposure to active social influences, and positive attitudes of drug use by healthcare professionals. Psychological factors included professional invincibility and protective beliefs. One logistic regression model assessed predictors of any alcohol and drug use versus no alcohol and drug use. A second model assessed risk factors predicting the top 25% of respondents based on total past year alcohol and other drug use, assumed to be at greatest risk for substance abuse or dependence. Therefore a composite of drug and alcohol use was developed in order to distinguish the HPs most heavily, using drugs and alcohol. Logistic regression, ANOVA and bivariate correlation examined potential bias from non-response which was not significant.

## Results

### Response

After accounting for non-deliverable surveys, the overall response rate was 68.7% (697/479) after all three waves of the survey. The highest response rate was from nurses (73.3%) and the lowest from physicians (63.4%). Except for a slight over-representation of men in dentistry and under-representation of men in pharmacy, the response from the survey was consistent with demographic information provided by the state. The mean age for the entire sample was 47.5 years (SD = 12.5). The age of the sample ranged from 42.3 years (SD = 10) for nurses, to 51.0 years (SD = 13.5) for dentists. The sample was mostly White, 92.9%, Non-Hispanic, 92.2%, and married 77.7%. Of dentists, 57.5 % reported being a Doctor of Dental Medicine (DMD) and 42.5% reported they were a Doctor of Dental Science (DDS). Most nurses (45.7%) reported they had graduated from either a diploma program or with an associate's degree, which were combined as pre-B.S., RNs. Another third (33.3%) indicated they had obtained a bachelor's degree, 20.9% reported they had obtained a master's degree and no one who responded had obtained a doctorate. Most pharmacists who responded, indicated that they had graduated with a BS in Pharmacy (75.2%), 9% had obtained a masters degree, 14.2% a PharmD and another 1.5% a Ph.D. Most physicians (96.2%) who responded to the survey indicated they were allopathic physicians (MD) and a few (3.8%) reported they were osteopathic physicians (DO).

The outcome measure consisted of a combination of past year drug/medication and past year alcohol use. The outcome measure was marginally skewed (2.1) and slightly kurtotic (4.4), but was acceptable without transformation [27]. This measure was then divided into two dichotomous groups that consisted of either no drug or alcohol use (n = 167) and any drug or alcohol use (n = 312) and used for "Model 1." To examine alcohol and drug use at extreme ends of the continuum, and provide an outcome measure of substance misuse, the alcohol and drug use groups were further subdivided into two orthogonal groups on the basis of use: the bottom 75% (n = 252) and the top 25% (n = 60) of respondents. In "Model 2" also called a "contingency ratio model," the bottom 75% were coded as 0 and the top 25% coded as 1. This scale therefore was sensitive to substantial, by comparison, alcohol use [M = 412.6 drinks per year, SD = 240.5], drug/medication use or a modest amount of use of all substances combined. It was therefore hypothesized that the top 25% of respondents would represent those encompassing the most alcohol and drug/medication involvement in this sample. The substance use variable without the non-users was normally distributed. Therefore, regressing the independent variables on these two models should provide a varied perspective on two unique levels of the summative use of past year alcohol and drugs/medications.

The measures taken together with the concordance rates of almost 67.5% [Model 1; n = 479, R^2 ^= .24, χ^2^(5) = 78.46, p = .0001] and 79.8% [Model 2; n = 312, R^2^= .41, χ^2^(6) = 98.42, p = .0001], suggest the predictors did a sound job of predicting AOD use. The difference between the full and reduced models for Model 1, χ^2 ^(6) = 10.36, p > .05, and Model 2, χ^2 ^(5) = 4.5, p > .05, were not significant, therefore only the significant predictors associated with the more parsimonious reduced models were retained for further individual predictor analyses.

To derive the reduced model each predictor was tested individually and the non-significant predictors were eliminated. The significant predictors for Model 1 (no use and/or any alcohol and or drug/medication use) shown in Table 1 include professional invincibility, χ^2 ^(1) = 8.09, p < .01, a moderate or more pattern of alcohol use, χ^2 ^(1) = 13.3, p < .001, professional/social and/or pharmaceutical offers for alcohol and other drugs, χ^2 ^(1) = 12.44, p < .001, as well as social networks that involve other known acquaintances with alcohol, drug or medication use problems, χ^2 ^(1) = 11.42, p < .001.

**Table 1:** Predictors for Any Past Year (Model 1) and Significant Alcohol and Drug Use (Model 2)

Predictors (IVs)	Model 1 (*N *= 479)				Model 2 (*N *= 312)			
	B (*SE*)	*β*	Odds Ratio (*OR*)	C.I.	B (*SE*)	*β*	Odds Ratio (*OR*)	C.I.

**Background Variables**								
Year licensed	-	-	-		.58* (.33)	-.21	.47	.3 – .9
Past month cigarette use	-	-	-		1.11^(.59)	.16	3.04	1.0 – 9.6
Pattern of alcohol use	3.01*** (.74)	.64	20.27	4.8–86.4	3.37*** (.51)	.79	29.01	10.6–79.6
**Psychological Variable**								
Professional invincibility	-1.43** (.52)	-.17	.24	.1 – .7	-	-	-	
**Social Variables**								
Professional/social and/or pharmaceutical offers for alcohol and or drug use	1.24*** (.26)	.28	3.45	1.1–2.8	-	-	-	
Social circles with known alcohol and drug abusers	.57*** (.24)	.15	1.77	2.0 – 5.8	-.66* (.33)	-.17	.52	.3–1.0
*Intercept*	*-.51 (.26)*				*-.51 (.38)*			

^p = .056, *p ≤ .05, **p ≤ .01, ***p ≤ .001.

*Notes: *Only significant predictors shown; - = not-significant; Gender, access to drugs in Model 1 and mean income, family history of substance use and religious affiliation in Model 2, were trending toward significance but removed from the models. C.I. = Confidence Interval. IVs = Independent Variables

Model 2 shown on the right in Table 1 displays the significant predictors for any versus significant AOD use groups. The regression coefficients suggest the net effects of all other variables, year licensed, χ^2 ^(1) = 5.5, p < .05, pattern of alcohol use, χ^2 ^(1) = 43.00, p < .001, and social networks, χ^2 ^(1) = 4.02, p < .05, were statistically significant predictors. There was also a strong trend toward significance for current cigarette use, χ^2 ^(1) = 3.6, p = .05. Finally several predictors trended toward significance but were excluded from both models (see Notes in Table 1).

## Discussion

Consistent with literature suggesting the prevalence of substance use for the general population declines with age after peaking in young adulthood [32], older HPs were at half the risk to report using significant levels of AOD use. Most studies of HPs in treatment suggest an age for substance related impairment to be middle adulthood and HPs younger than 35 tend to abuse a combination of AOD [33]. Knowing that younger HPs are at the most risk would suggest that professional licensing boards have an opportunity to broach the subject of non-prescribed drug use with newly licensed HPs.

The possible relationship between cigarettes and AOD use is consistent with previous research [34]. Current cigarette use was shown to be a significant predictor of high-risk alcohol use in an ambulatory setting [35] and a strong correlate to AOD use in college students [36]. Additionally, evidence suggests that cigarette smoking is a significant risk factor for opioid dependence [37], a drug class of major concern for HPs [15]. Part of the weakness attributable to this trend toward an association with alcohol and other drug use lies with the low base rate of cigarette smoking in general in the healthcare professionals surveyed in this study (n = 18). Without drawing causal conclusions however, it may be prudent to intervene with HPs who continue to smoke despite the obvious health risks.

A moderate or more pattern of alcohol use was a significant predictor for any drug use as well as significant drug use. Though the link between alcohol and other drug use is clearly established, that that pattern of alcohol use was so strongly associated with an increased risk of alcohol and other drug use in HPs, however was notable. Alternatively such a strong relationship may also be the result of modest multicollinearity between the two variables (r = .67).

HPs who strongly disagreed they needed drugs to work were at significantly less risk to report AOD use during the past year. Previous findings suggest a strong belief in the immunity to becoming addicted to drugs by some HPs that may be instrumental toward decision making to use drugs [7]. The current study suggests that those who would not take drugs for instrumental purposes, such as to stay at work, were less likely to use a substance of any kind. Contrary to what was expected, however this finding suggests that pharmaceutical invincibility may not be a false belief but a valid and real belief system for HPs, being especially protective in a no use versus use paradigm.

Social circles and pharmaceutical offers of AOD use was a significant predictor for the no use versus use model only. Perhaps social contacts are important for substance use in general, but not at a significant level of use. Potentially, normative use of AOD may be facilitated by social relationships whereas significant use may be characterized by withdrawing from social relationships. Supporting this notion is that the odds ratios for knowing others who are substance abusers (social circles) between the two models vary from increasing one's chances of any past year substance use by over three-quarters to decreasing one's risk for significant substance use by one-half. Such a contrast is consistent with previous findings noting that substance-impaired HPs are often loners and become isolated [38]. There is no healthcare professional drug culture. Healthcare professionals may limit their contact with others out of fear of discovery and rigorously deny problems to themselves or others.

This study is limited in several ways. Primarily, the composite used to combine alcohol and drug use as a variable did not allow analysis of the drug and alcohol use by the top 25% of HPs. These results are most likely affected by geographic location and may not generalize to areas outside of the northeast. A national survey using similar methodology would help address this bias associated with regional samples. Additionally, the data presented are probably a conservative estimate of AOD use by the respondents in the state. Healthcare professionals are probably uniquely motivated to under-report drug use in particular, in light of the fear of the legal repercussions. The response rate of almost 69% means that over 31% of respondents did not respond and could contribute to measurement error due to non-response. However, considering the sensitive topic, the response rate for the survey was strongly commensurate with the other studies of HPs previously noted. Alternatively, this sample was drawn from a single state, limiting the generalizability to the greater population of HPs. Moreover, the alcohol pattern measure used in the present study, which was a subjective measure of alcohol use frequency, was as previously noted, potentially a less specific and broader measure of alcohol use as well as non-use, resulting in increased power as a predictor. Recent evidence suggests that a single question assessing one's pattern of alcohol use may underestimate alcohol use [39]. This measure contrasts with the uni-dimensional but more precise quantity-frequency index also used in the current study, but was a non-significant predictor. In concert with the possibility that total amount of alcohol consumed may not be a useful predictor of alcohol-related problems [40] surveys of substance use have found [41] that respondents may report less alcohol (and other substances) use resulting in an underestimate of alcohol use; relative to the broader pattern measure used in the current study. On the other hand it is also possible the relationship between a pattern of drinking is quite predictive of perceived problems as Wood, et al. [42] demonstrated that non-daily drinkers scored higher on the Alcohol Dependence Scale than daily drinkers, putatively because non-daily drinkers perceived intoxication as more impairing due to low tolerance associated with their pattern of drinking. This would putatively explain the strong relationship between perceived alcohol use considered more than appropriate and pattern of use, relative to the broad spectrum of primarily non-daily drinking habits of the HCP population.

Despite the limitations, these findings indicate a need to educate HPs and students regarding substance use and abuse. People entrust their personal welfare and safety to those in the health professions. The profession in turn has an ethical obligation to ensure that its practitioners can discharge their duties with skill and safety. Policies should promote early discovery of professionals who overuse AOD in order to minimize the period of time that patients are at risk of being harmed.

Various healthcare professional organizations have worked to shape policy with regard to substance use in general. For example, the Physician's Leadership on National Drug Policy [43] is an ad hoc group of physicians with experience in health, medical care, and policy development. In June 1997, this group developed and adopted a Consensus Statement on national drug policy toward illegal drugs that placed a new emphasis on national drug policy by refocusing on the prevention and treatment of harmful drug use. This requires reallocating resources toward drug treatment and prevention, utilizing criminal justice procedures which are shown to be effective in reducing supply and demand, and reducing broad regulations of addiction treatment programs [43].

More specifically, the American Pharmacists Association [44] as well as American Association of Colleges of Nursing [45] have worked to adopt and institute policies of treatment, prevention, and rehabilitation of AOD-using healthcare professionals. Agencies such as the Drug Enforcement Administration, state departments of health, medical boards, state medical associations, and colleges teaching healthcare professional programs, should also coordinate discussions among HPs related to AOD use. Dissemination of information relating to the warning signs of drug abuse and sources of help for those affected by AOD should be continual goals of these agencies. In particular, educational points to highlight include such factors as the hazards of self-treatment with prescription drugs no matter how minor or infrequent; alcohol use, though modest, may still be an initiate of concomitant substance use by HPs; addiction education is a priority need for HP students and professionals and confidential treatment exists and works well for the majority of addicted HPs [46].

## Authors' contributions

GK performed the study, initial analysis and write-up. DL was involved with evaluation of the analysis, the initial write-up and revised drafts of the manuscript.
